# Inflammatory and degenerative phases resulting from anterior cruciate rupture in a non‐invasive murine model of post‐traumatic osteoarthritis

**DOI:** 10.1002/jor.23872

**Published:** 2018-03-14

**Authors:** Sophie J. Gilbert, Cleo S. Bonnet, Paulina Stadnik, Victor C. Duance, Deborah J. Mason, Emma J. Blain

**Affiliations:** ^1^ Arthritis Research UK Biomechanics and Bioengineering Centre, Biomedicine Division, School of Biosciences Cardiff University Museum Avenue Cardiff CF10 3AX UK

**Keywords:** post‐traumatic osteoarthritis, mechanical load, non‐invasive mouse model, inflammation, degeneration

## Abstract

Joint injury is the predominant risk factor for post‐traumatic osteoarthritis development (PTOA). Several non‐invasive mouse models mimicking human PTOA investigate molecular mechanisms of disease development; none have characterized the inflammatory response to this acute traumatic injury. Our aim was to characterize the early inflammatory phase and later degenerative component in our in vivo non‐invasive murine model of PTOA induced by anterior cruciate ligament (ACL) rupture. Right knees of 12‐week‐old C57Bl6 mice were placed in flexion at a 30° offset position and subjected to a single compressive load (12N, 1.4 mm/s) to induce ACL rupture with no obvious damage to surrounding tissues. Tissue was harvested 4 h post‐injury and on days 3, 14, and 21; contralateral left knees served as controls. Histological, immunohistochemical, and gene analyzes were performed to evaluate inflammatory and degenerative changes. Immunohistochemistry revealed time‐dependent expression of mature (F4/80 positive) and inflammatory (CD11b positive) macrophage populations within the sub‐synovial infiltrate, developing osteophytes, and inflammation surrounding the ACL in response to injury. Up‐regulation of genes encoding acute pro‐inflammatory markers, inducible nitric oxide synthase, interleukin‐6 and interleukin‐17, and the matrix degrading enzymes, ADAMTS‐4 and MMP3 was detected in femoral cartilage, concomitant with extensive cartilage damage and bone remodelling over 21‐days post‐injury. Our non‐invasive model describes pathologically distinct phases of the disease, increasing our understanding of inflammatory episodes, the tissues/cells producing inflammatory mediators and the early molecular changes in the joint, thereby defining the early phenotype of PTOA. This knowledge will guide appropriate interventions to delay or arrest disease progression following joint injury. © 2018 The Authors. *Journal of Orthopaedic Research*® Published by Wiley Periodicals, Inc. on behalf of the Orthopaedic Research Society. J Orthop Res 36:2118–2127, 2018.

Unlike primary/idiopathic osteoarthritis (OA), the predominant risk factor for development of secondary, often referred to as post‐traumatic osteoarthritis (PTOA) is joint injury. Approximately 12% of all diagnosed OA incidents likely arise from a previous joint trauma,^1^ with a major joint injury resulting in approximately 25% of OA cases in susceptible joints, for example, knee, ankle,^2^ and >50% of patients with an anterior cruciate ligament (ACL) injury showing radiographic evidence of OA 10–12 years post‐injury.^3^, ^4^

Various surgical models of PTOA e.g. destabilization of the medial meniscus (DMM), meniscal transection (MNX), are unsuitable for detecting early changes at the time of injury due to acute inflammatory responses resulting from the surgery itself. Several non‐invasive mouse models mimicking human PTOA have been established to investigate molecular mechanisms that underpin disease development and test interventions immediately post‐injury. These models are reliant on application of a defined, externally controlled mechanical insult/injury predisposing the joint to degeneration akin to that observed in the human disease.^5^, ^6^, ^7^ Non‐invasive mouse PTOA models initiate joint degeneration, although the method of mechanical induction varies between each, ranging from the very earliest model of intra‐articular fracture of the proximal tibia,^8^ a single compressive load that applies an acute injurious impact to the knee,^9^, ^10^ to cyclic tibial compression of the lower leg.^11^, ^12^, ^13^, ^14^ As these PTOA models apply different magnitudes and frequencies of load to the limb, and elicit subtle variances in severity of joint pathology induced, Blaker *et al*. proposed a classification system based on an “idealised force‐displacement curve” with three categories referred to as Types I–III PTOA.^5^ Type I PTOA models reflect immediate structural damage and/or joint instability induced by mechanical overloading to instigate ACL rupture or intra‐articular fracture. Type II PTOA models involve application of a significant mechanical insult, but unlike those of Type I PTOA, do not result in major structural defects to the joint tissues nor observable joint instability, for example, single or multiple sessions of joint loading, single sub‐failure joint loading, or loads that induce only partial soft tissue tears. In contrast, Type III PTOA is characterized by low‐level joint loading which affects tissue homeostasis but does not impact on joint stability.

Type I PTOA models of ACL rupture, typically induced by a single episode of compressive load to the lower limb (12N, 1 mm/s), are characterized by extensive loss of trabecular bone, osteophyte formation and loss of articular cartilage proteoglycans, concomitant with fissuring and chondrocyte apoptosis in the superficial zone over an 8‐week period post‐induction of injury.^9^ Increasing the loading rate from 1 mm/s in this model, which induces an avulsion fracture,^9^ to 500 mm/s which results in a mid‐substance tear, also leads to substantial joint degeneration and an OA phenotype.^15^ Alternatively, multiple cycles of tibial compressive load have also been used to rupture the ACL and damage the articular surface resulting in PTOA with similarities to human disease.^14^ Type I PTOA, caused by ACL rupture, generates a significant inflammatory response; it is thought that PTOA progression is correlated with the inflammatory profile as opposed to the sole contribution of one mediator.^16^ Following mechanical insult, inflammatory mechanisms are hypothesized to instigate development and progression of joint degeneration including cellular infiltration, production of cytokines and chemokines, release/activation of proteolytic enzymes, chondrocyte apoptosis, subchondral bone remodeling including osteophyte production, synovial fibrosis and activation of other pathways that mediate tissue catabolism.^17^, ^18^ Some of these biological effects instigated at the time of trauma, can propagate a sustained response, weeks to months post‐injury and are thought to contribute to PTOA development.^19^

Surprisingly, there is limited detail on the inflammatory response to acute traumatic injury, with reporting of the presence of synovitis only (type II PTOA model:^13^ Type I PTOA model:^14^). Furthermore, the inflammatory response in PTOA models has not been characterized from the immediate early events through to late stage disease. An advantage of non‐invasive mechanical injury models is that they allow characterization of very early events that directly arise from the trauma itself, on a scale of hours to days, as well as the extensive joint degeneration observed over time. Since these early inflammatory events may drive end stage joint degeneration, such models provide an opportunity to define the “window of therapeutic opportunity” and identify targets to delay or arrest the development of PTOA.

The aim of this study was to characterize the inflammatory and degenerative phases as well as the later degenerative component in our in vivo murine model of type I PTOA, where a single compressive load ruptures the ACL. Definition of the inflammatory episodes, the tissues/cell types producing inflammatory mediators and the early molecular changes in the joint in the early phenotype of PTOA will guide appropriate interventions to delay or arrest disease progression following joint injury.

## METHODS

All chemicals were from Sigma unless otherwise stated and were of analytical grade or above. Primary antibodies: Rat monoclonal to F4/80 (clone Cl:A3‐1; AbD Serotec®, Biorad, Kidlington, UK), rabbit monoclonal to Cd11b (clone EPR1344; Abcam, Cambridge, UK), rabbit polyclonal to IL‐6 (Novusbio Biotechne, Abingdon, UK), Rabbit polyclonal to IL‐17A (Peprotech, London, UK).

### Animals

Procedures were performed in compliance with the Animals (Scientific Procedures) Act 1986 [Home Office licence 30/2959] according to Home Office and ARRIVE guidelines.^20^ Twelve‐week old male C57Bl6 mice (∼25 g, Charles River, UK), randomly assigned to either experimental or control groups, were randomly allocated to MB1 cages (960 cm^2^) in groups of 5 (12 h light–dark cycles, ad libitum food and water). Loading was performed in the morning. All mice received Buprenorphine (0.05 mg/kg) subcutaneously at the start of the experiment, moved freely throughout the experiment and were monitored for welfare, knee swelling (digital callipers) and lameness until the end of the experiment. Animal numbers for each measurement are shown (Suppl. Table S1).

### Induction of PTOA

Mice were anaesthetized with isoflurane and custom built cups^21^ used to hold the right ankle and knee in flexion with a 30 degree offset prior to the application of a 0.5N pre‐load (ElectroForce® 3200, TA Instruments, Elstree, UK). A single 12N load at a velocity of 1.4 mm/s was then applied resulting in ACL rupture. These parameters were chosen based upon prior experiments showing that 12N was a threshold force in these mice where a mid‐substance tear of the ligament would occur immediately on application of one load cycle at 1.4 mm/s. ACL rupture was identified through the waveform as a continued increase in displacement following release of the applied compressive force with an audible “popping” sound. To determine whether contralateral knees could serve as unloaded controls, they were compared with the right and left knees of naïve mice which were not subjected to anaesthetic or load.^22^ Mice were culled by cervical dislocation at multiple time points to assess for the presence of an inflammatory response and joint degeneration.

### Specimen Preparation

For histology and immunohistochemistry, hind limbs, at an orientation of 90 degrees, were immediately fixed post‐mortem in formalin (2 days, 10% neutral buffered formalin), decalcified for 2 weeks (4°C, 10% EDTA, Fisher Scientific, Loughborough, UK), and either embedded frontally in paraffin blocks for coronal sectioning parallel to the tibia, or embedded side on for sagittal sectioning. Serial sections (5 μm) obtained at 100 μm intervals through the joint, were dewaxed and rehydrated prior to staining with Haematoxylin and Eosin, Toluidine Blue or processing for immunohistochemistry. For x‐ray analysis, knee joints were stored in 70% ethanol following formalin fixation. Histology on sagittal sections and x‐ray analysis (KODAK In‐Vivo Imaging System FX Pro AMV, Lincoln, UK)^23^) confirmed rupture with no obvious damage to the synovium, menisci or other joint tissues (Suppl. Fig. S1) as previously reported for other non‐invasive mouse models that recapitulate isolated ACL ruptures.^9^, ^24^

### Histological Scoring

The medial femoral condyle (MFC), medial tibial plateau (MTP), lateral femoral condyle (LFC), and lateral tibial plateau (LTP) from 2 to 4 coronal sections (either side of the centre of the joint, approximately 200 µm apart) were scored for sub‐synovial inflammation and degenerative changes by two or four independent observers, respectively, blinded to treatment. For each mouse, a single score representing the mean value from all observers and sections was used for statistical comparison. The Osteoarthritis Research Society International (OARSI) score (Glasson et al.^25^; Suppl. Table S2) was used with Toluidine Blue stained sections (0–6 for osteoarthritic changes, 0–3 for subchondral bone changes, and 0–5 for proteoglycan depletion giving a total score out of 14 for each quadrant). In addition, the presence (+1) or absence (0) of osteophytes within the joint was noted. Haematoxylin and Eosin sections were scored for the presence of sub‐synovial inflammation (Nowell et al.^26^; Suppl. Table S2).

### Immunohistochemistry

Consecutive coronal sections were deparaffinised and rehydrated prior to antigen retrieval (1 mg/ml trypsin for 1 h at 37°C). Each subsequent step was performed at room temperature unless stated otherwise and between each incubation step, sections were washed 3 × 5 min in 0.01 M phosphate buffered saline (PBS, pH7.4) containing 0.1% (v/v) Tween®20 (wash buffer). All antibodies were diluted in wash buffer (F4/80 1:250, Cd11b 1:100, IL‐6 1:500, and IL‐17A 1:100). Endogenous peroxidase activity was blocked with 0.3% (v/v) hydrogen peroxide for 30 min. Sections were subsequently treated with 10% normal goat serum for 1 h prior to overnight incubation (4°C) with primary antibody, rabbit or rat IgGs or PBS. Sections were subsequently incubated for 30 min with biotinylated anti‐rabbit or anti‐rat secondary antibodies, developed with nickel enhanced diaminobenzidine (DAB) (Vectastain® Elite ABC kit, DAB, Vector Laboratories, Peterborough, UK) and finally dehydrated, cleared in xylene and mounted. Slides were viewed on a Leica DMRB microscope. IgG and PBS controls were negative (Suppl. Fig. S2).

### Quantitative RT‐PCR Analysis of Gene Expression

Four hours after ACL rupture, mice were culled and knee joints immediately dissected to expose the femoral condyle cartilage. Using forceps, pressure was applied to the top of the femur to “pop” the cartilage from underlying subchondral bone at the tidemark. Cartilage was pooled from injured (*n* = 9) or uninjured (*n* = 9) contra‐lateral knees and immediately snap frozen in liquid nitrogen prior to RNA extraction using TRIzol® reagent according to manufacturer's protocol (ThermoFisher Scientific). Total RNA was purified using a mirVana™ miR Isolation Kit followed by DNAse treatment (Ambion, Fisher Scientific, Loughborough, UK) following the manufacturer's protocol and assessed using a spectrophotometer (Nanodrop 1000, ThermoFisher Scientific, Stockport, UK) and 2100 Bioanalyzer (Agilent Technologies) with A260/280 values between 1.8–2.0 and RIN scores >8. Complementary DNA (cDNA, 20 μl total volume) was generated from 300 ng total RNA using SuperScript® III reverse transcriptase (Invitrogen TM, Fisher Scientific, UK) and 0.5 μg random primers (Promega, Southampton, UK) according to manufacturer's instructions, and 1 μl cDNA utilized in each qPCR assay. Quantitative polymerase chain reaction (qPCR) was performed using SYBR green detection (Brilliant III Ultra‐Fast SYBR® QPCR mix, Agilent Technologies) using intron‐spanning primers for genes of interest (Suppl. Table S3^27^), on a QPCR machine (MxPro3000, Agilent Technologies). All reactions were carried out at an annealing temperature of 60°C unless specified otherwise (Suppl. Table S3) cycling conditions were: 95°C‐3 min (1 cycle), 95°C‐15 s followed by 60°C‐30 s (40 cycles), 95°C‐1 min followed by 60°C‐30 s followed by 95°C‐30 s (1 cycle). Primers (MWG‐Biotech AG, Germany), were used at a final concentration of 200 nM and validated using cDNA standard curves with all primer efficiencies between 90 and 110%.^28^ Data were normalized to 18s and β‐actin which were identified from 8 reference genes using RefFinder (http://150.216.56.64/referencegene.php) as maintaining stable expression under these experimental conditions.^27^ Fold‐change in expression of genes of interest was calculated using the 2^−ΔΔCT^ method,^29^ after normalization to the reference genes and presented relative to the uninjured contra‐lateral limb.

### Data Analysis

Data is presented as mean ± standard error mean (SEM). Data were tested for normality and equal variances and transformed if necessary prior to testing by general linear model analysis of variance (GLM ANOVA) and Tukey's post hoc test (Minitab 16). Differences were considered significant at *p* ≤ 0.05.

## RESULTS

### Sub‐Synovial Inflammation Results From ACL Injury

**Figure 1:**
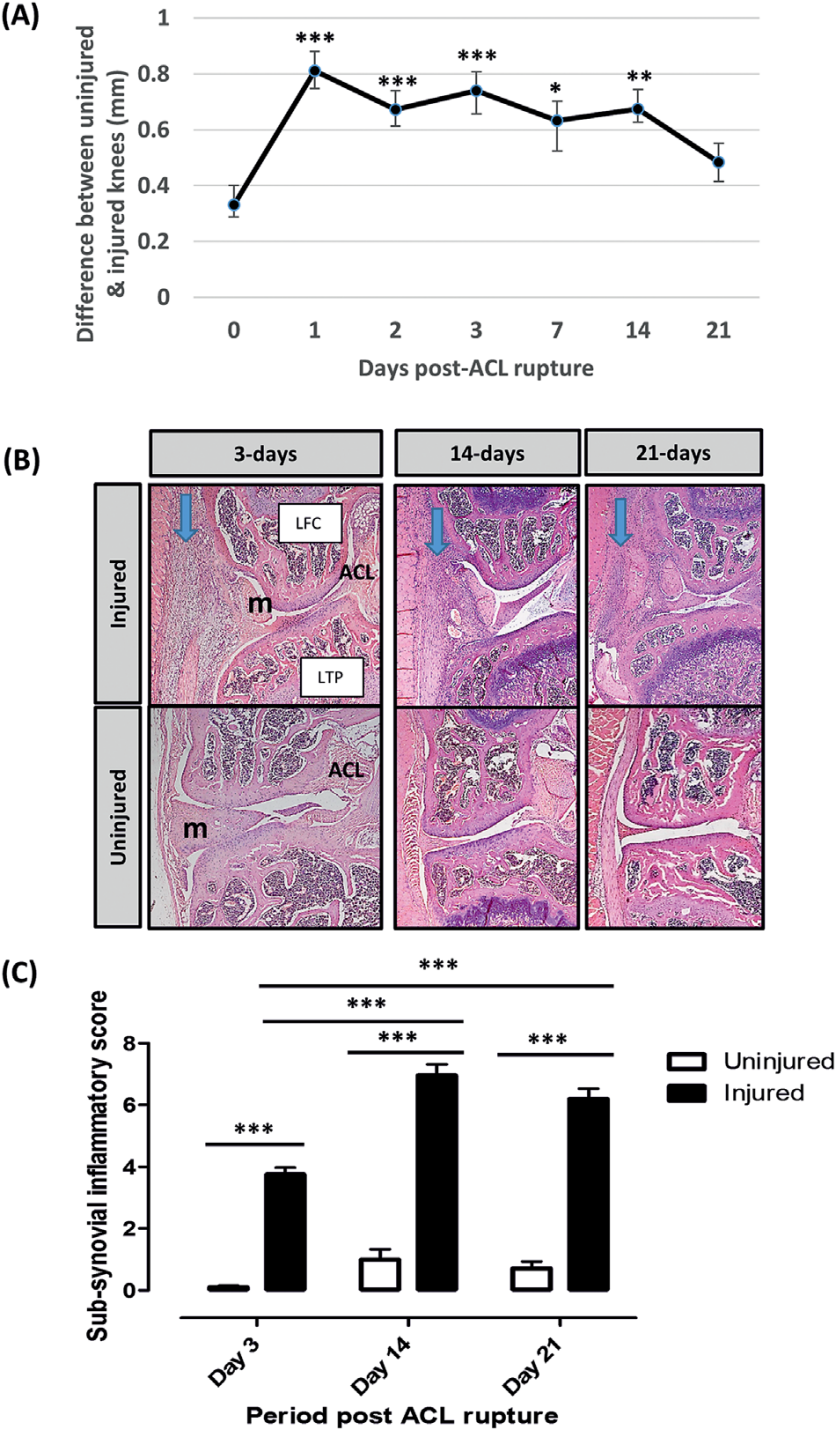
Knee joint swelling was measured by digital callipers and expressed as the difference in size between the uninjured and injured leg. *p < 0.05, **p < 0.01, ***p < 0.001 GLM ANOVA versus day‐0 (A). Coronal sections from uninjured and injured legs over the 21‐day time course were stained with Haematoxylin and Eosin (B). Cellular infiltrate in the sub‐synovial lining is indicated (arrows). Whole joints were scored for the presence of sub‐synovial inflammation (C). LTP, lateral tibial plateau; LFC, lateral femoral condyle; m, meniscus. *** GLM ANOVA (*p* < 0.001).

Knee swelling following ACL rupture peaked at day‐1 and slowly receded over the 21‐days (Fig. 1A). Cellular infiltrate in the sub‐synovial lining was evident over the 21‐days (Fig. 1B) and the extent of sub‐synovial inflammation quantified^26^ (Fig. 1C). By day‐3 there was a significant increase in inflammation score in the injured legs compared with the uninjured legs (3.75 ± 0.23 mm vs. 0.1 ± 0.06 mm; *p* < 0.001). The level of inflammation peaked in injured knees at day‐14 (6.97 ± 0.36 mm vs. 0.99 ± 0.33 mm; *p* < 0.001, *p* < 0.001 vs day‐3 injured legs) and remained high at day‐21 (6.57 ± 0.33 mm vs 0.43 ± 0.22 mm; *p *< 0.001 vs uninjured left leg, *p *< 0.001 vs day‐3 injured legs). There was no statistically significant change in the level of inflammation in the uninjured legs at any time point.

### Mature and Inflammatory Macrophages Increase Following ACL Injury

**Figure 2:**
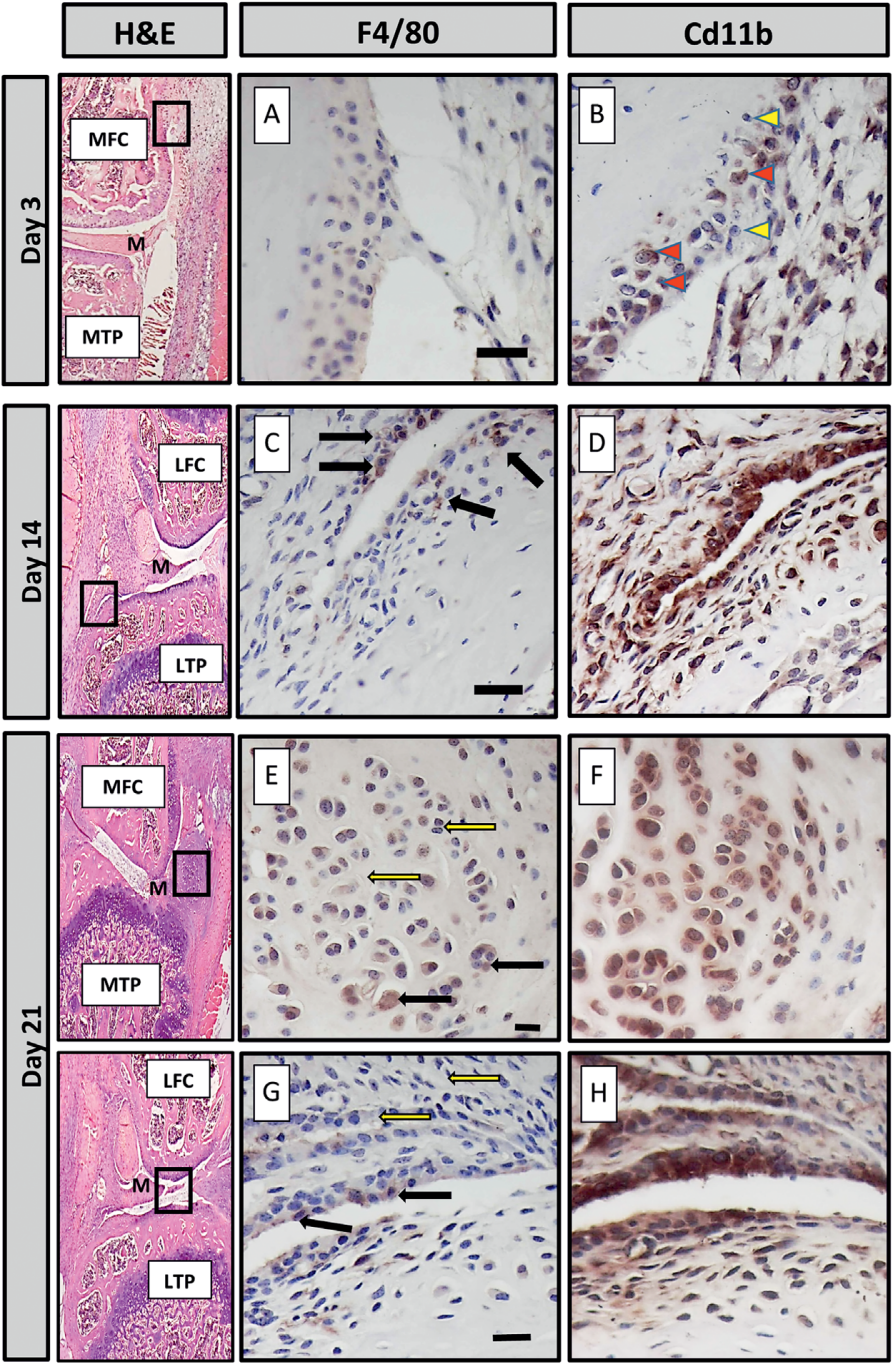
The localization of F4/80 and CD11b in consecutive sections taken from injured knees of mice culled at 3, 14, and 21‐days post‐ACL rupture was determined by immunohistochemistry. Haematoxylin and Eosin (H&E) stained sections indicate where magnified images are derived from. At day‐3 (A and B), staining for F4/80 was very weak whereas CD11b was found in a large number of cells within the inflammatory infiltrate (solid arrowheads); some cells remained negative (white arrowheads). At day‐14 (C and D) and ‐21 (E and G), a limited number of F4/80^+^ cells were located within the synovial infiltrate (black arrows); many cells remained negative (white arrows). Cells within the developing osteophytes (E) were also positive. CD11b cells were located throughout the developing osteophyte (F) and synovial infiltrate. LTP, lateral tibial plateau, LFC, lateral femoral condyle; MTP, medial tibial plateau; MFC, medial femoral condyle, m, meniscus. Scale bar = 20 μM.

Cells positive for F4/80 and CD11b as markers of mature and inflammatory macrophages respectively, were detected by immunohistochemistry (Fig. 2). Three days after ACL rupture cells expressed low or no F4/80 (Fig. 2A), but 14 days after injury the abundance of F4/80^+^ cells increased within the infiltrate (Fig. 2C, black arrows). In addition, at day‐21, cells within the developing osteophytes expressed F4/80 (Fig. 2E) although many cells of the infiltrate and some cells in the osteophyte were negative (yellow arrows). CD11b expressing cells were present within the sub‐synovial cellular infiltrate 3‐days after ACL rupture (Fig. 2B), with their abundance in the infiltrate increasing by day‐14 (Fig. 2D) when they were also detected in the developing osteophyte (data not shown). Cells expressing CD11b were also detected in the infiltrate and developing osteophyte at day‐21 (Fig. 2F). Cells expressing CD11b were present within the inflammatory infiltrate surrounding the ACL/posterior cruciate ligament (PCL) complex from day‐3 onwards and within the ACL/PCL at day‐14 and ‐21 (Suppl. Fig. S3). These expression patterns were not observed in uninjured legs as no inflammatory infiltrate or osteophytes were present (data not shown). In both uninjured and injured legs, osteoblasts, bone marrow, and megakaryocytes expressed F4/80 and cells within the blood vessels expressed CD11b (Suppl. Fig. S3).

### IL‐6 and IL‐17A Protein Expression Increases in Injured Knees

**Figure 3:**
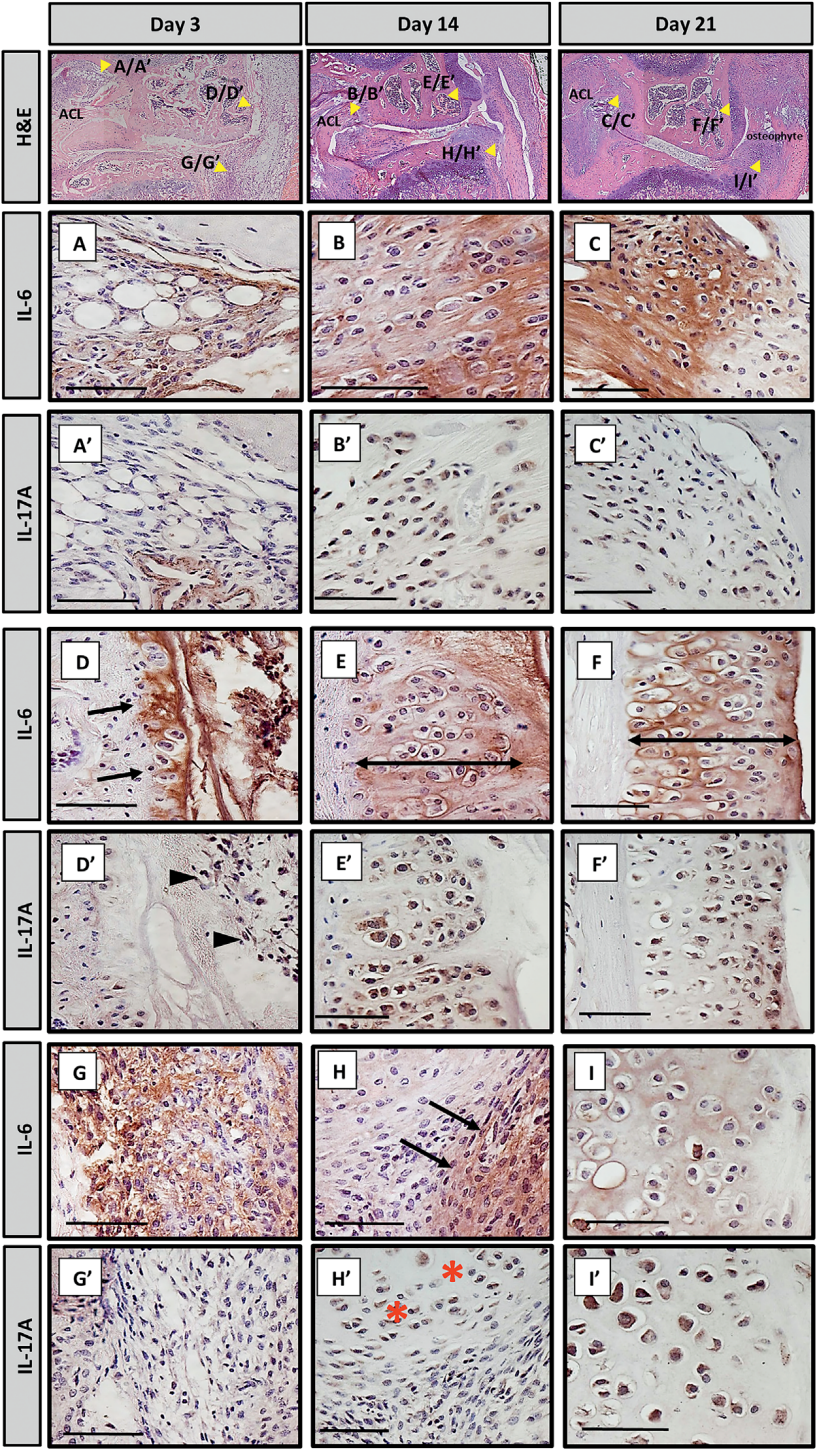
Consecutive sections were taken from injured knees of mice culled at 3, 14, and 21‐days post‐ACL rupture and stained for IL‐6 and IL‐17A by immunohistochemistry. Haematoxylin and Eosin (H&E) stained sections indicate where magnified images are derived from. Medial compartment inflammation is shown. Strong staining for IL‐6 was observed within the extracellular matrix of the ACL ligament at day‐3 (A), day‐14 (B), and day‐21 (C). In contrast, at day‐3, no staining for IL‐17A was found within the ligament apart from surrounding a blood vessel (A’). At day‐14 (B’) and day‐21 (C’), IL‐17A was detected within the cells of the ligaments. At day‐3 the periosteum of the femoral condyle (arrows, D) was positive for IL‐6 but negative for IL‐17A (D’); intracellular staining in the inflammatory infiltrate nearby was, however, present (arrowheads D’). At day‐14 and ‐21, IL‐6 was observed within the matrix surrounding the hypertrophic chondrocytes of the developing osteophytes (double headed arrow, E and F, respectively). Intracellular staining for IL‐17A was seen on consecutive sections (E’ and F’, respectively). At day‐3 there was abundant staining for IL‐6 within the matrix surrounding the synovial infiltrate (G); consecutive sections were negative for IL‐17 (G’). At day‐14, IL‐6 was still found within this region (H); IL‐17A was absent but present within the nearby meniscus (H’, *). At day‐21, the matrix surrounding hypertrophic chondrocytes within the meniscus were positive for IL‐6 (I) and strong staining for IL‐17A was found within these cells (I’). ACL, anterior cruciate ligament. Scale bar = 50 μM.

Staining for IL‐6 and IL‐17A was carried out at 3‐, 14‐, and 21‐days post‐ACL rupture in sections from injured (Fig. 3) and uninjured knees (Suppl. Fig. S4). In uninjured knees, IL‐6 was expressed in cells surrounding the ACL/PCL complex and within the bone, the growth plates and synovium, and IL‐17 in chondrocytes, osteoblasts, and growth plates. In injured knees, IL‐6 expression was abundant within the matrix of the ACL ligament at all time points (Fig. 3A–C) whereas IL‐17A was not expressed within the ACL at day‐3 (Fig. 3A’) but was at day‐14 and −21 (Fig. 3B’ and C’). Within the femoral condyle after injury, periosteal cells expressed IL‐6 but not IL‐17A at day‐3 (Fig. 3D and D’) although IL‐17A was expressed in the nearby inflammatory infiltrate (Fig. 3D’). At day‐14 and ‐21, IL‐6 immunolocalized to matrix and IL‐17A intracellularly to regions where hypertrophic chondrocytes of developing osteophytes resided (Fig. 3E, E’, F, F’). At day‐3 and ‐14 after injury, IL‐6 expression was abundant within the matrix surrounding the synovial infiltrate (Fig. 3G and H) whereas consecutive sections were negative for IL‐17A (Fig. 3G’ and H’). IL‐6 was not detected in the meniscus until day‐21 after injury (Fig. 3I), whereas IL‐17A was expressed within the meniscus at day‐14 and ‐21 (Fig. 3H’ and I’).

### Joint Degeneration Occurs Rapidly Following ACL Injury

**Figure 4:**
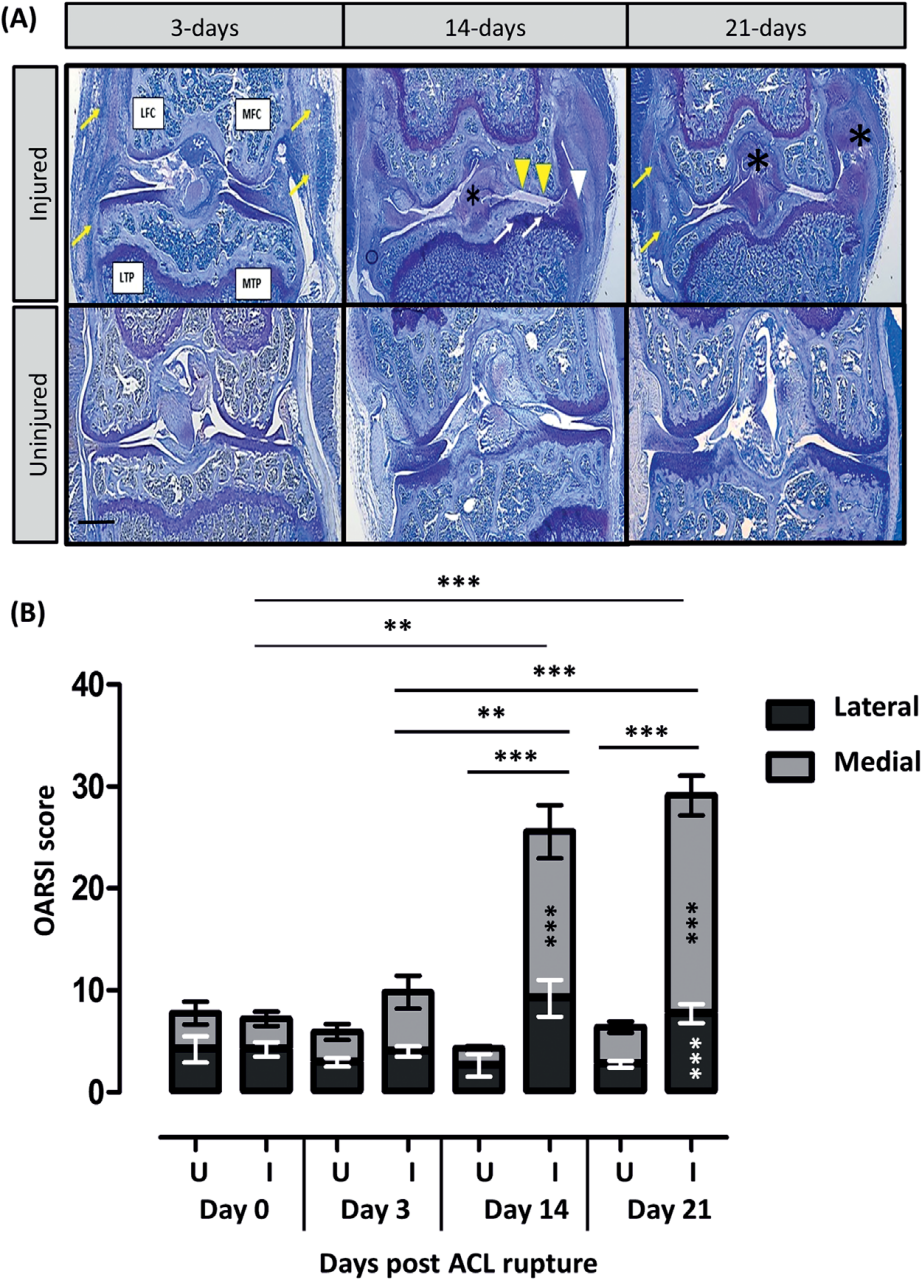
Toluidine blue stained coronal sections taken from knees at 3, 14, and 21‐days post‐ACL rupture (A). Injured knees are shown in the top panel and uninjured knees in the bottom panel. All four quadrants of the knee joint are indicated: Lateral and medial tibial plateaus (LTP, MTP, respectively), lateral and medial femoral condyle (LFC, MFC, respectively). Synovial thickening and increased cellular infiltrate (arrows), new matrix deposition within ACLs and collateral ligaments (*), significant medial compartment cartilage loss (arrowheads), bone remodelling (arrows) and osteophyte formation (arrowheads) are shown. Scale bar = 500 μM. Sections were scored for degeneration using the OARSI score (B–F). GLM ANOVA comparing the effect of time (days), uninjured (U) versus injured (I), medial versus lateral, femoral condyle vs tibial plateaus; *p < 0.05, **p < 0.01, ***p < 0.001. Total score increased with time (B) and at day‐14 and ‐21, the medial compartment OARSI score in the injured leg was significantly different to the uninjured leg ***p < 0.001; in addition, at day‐21, the lateral compartment OARSI score in the injured leg was significantly different to the uninjured leg ***p < 0.001. OARSI scores for the femoral condyle and tibial plateaus (C) and sub‐parameters, osteoarthritic changes (D), proteoglycan depletion (E), and subchondral bone (D). At day‐14 and ‐21, the tibial compartment OARSI score in the injured leg was significantly different to the uninjured leg ***p < 0.001; at day‐21, the femoral compartment OARSI score in the injured leg was also significantly different to the uninjured leg ***p < 0.001.

An acute inflammatory response, in the form of synovial thickening and increased cellular infiltrate, was observed at day‐3 following ACL rupture (Fig. 4A). By day‐14, new matrix deposition was observed within injured ACLs along with substantial medial compartment cartilage loss, bone remodeling, and osteophyte formation (Fig. 4A). Synovial thickening and cellular infiltrate remained at day‐21 following ACL injury as well as increased matrix deposition within the ACL and joint capsule. To assess whether ACL ligament injury caused joint degeneration, knees were scored using the OARSI score^25^ (Fig. 4B). No significant change was detected with the uninjured, contralateral legs across the time course and these did not differ from naive knees (data not shown), therefore contralateral knees were used for statistical comparison. GLM ANOVA of OARSI scores comparing time versus injury at day‐14 revealed significant effects of time (*p* < 0.001) and injury (*p* < 0.001). In the injured leg at day‐14, total OARSI score (sum of all three parameters and all four quadrants) was increased 5.9‐fold compared to uninjured legs (25.6 ± 3.2 vs. 4.3 ± 1.3; *p* < 0.001 following log transformation of data), and 3.4‐fold (7.4 ± 0.2; *p* = 0.002) and 2.6‐fold (9.8 ± 1.4; *p* = 0.006) compared with those of the injured leg at days‐0 and ‐3, respectively. At day‐21, total scores were 4.6‐fold higher in the injured leg compared to the uninjured leg (29.8 ± 2.5 vs. 6.5 ± 0.6; *p* < 0.001) and four‐fold and three‐fold higher than those of the injured leg at day‐0 (*p* < 0.001) and day‐3 (*p* < 0.001), respectively. There were no significant changes in the uninjured leg over time. At day‐14, the medial compartment was significantly more damaged in the injured compared to uninjured leg (*p *< 0.001; Fig. 4B). In contrast, at day‐21, damage within both the lateral and medial compartments contributed to the total OARSI score (*p* < 0.001 following ranking of data; Fig. 4B). The tibial plateau of the injured leg had a significantly increased OARSI score by day‐14 (15.7 ± 1.7 vs. 2.5 ± 0.1; *p* = 0.02), which did not significantly alter by day‐21, whereas the OARSI score only significantly increased within the femoral condyle at day‐21 (13.5 ± 1.6 vs. 2.5 ± 0.3; *p* < 0.001; Fig. 4C). Breaking down the OARSI score components revealed significant OA damage in the injured leg by day‐3 (*p* = 0.03), that increased on day‐14 (*p* < 0.001) and day‐21 (*p* < 0.001), and proteoglycan loss (*p* < 0.001) and subchondral bone changes (*p* = 0.002), which were significantly increased at day‐14, and did not alter significantly at day‐21 (Fig. 3D–F). Osteophytes were present in both the lateral and medial compartments in all injured knees from day‐14 onwards.

### Up‐Regulation of Genes Involved in Inflammatory and Degenerative Responses Following ACL Injury

**Figure 5:**
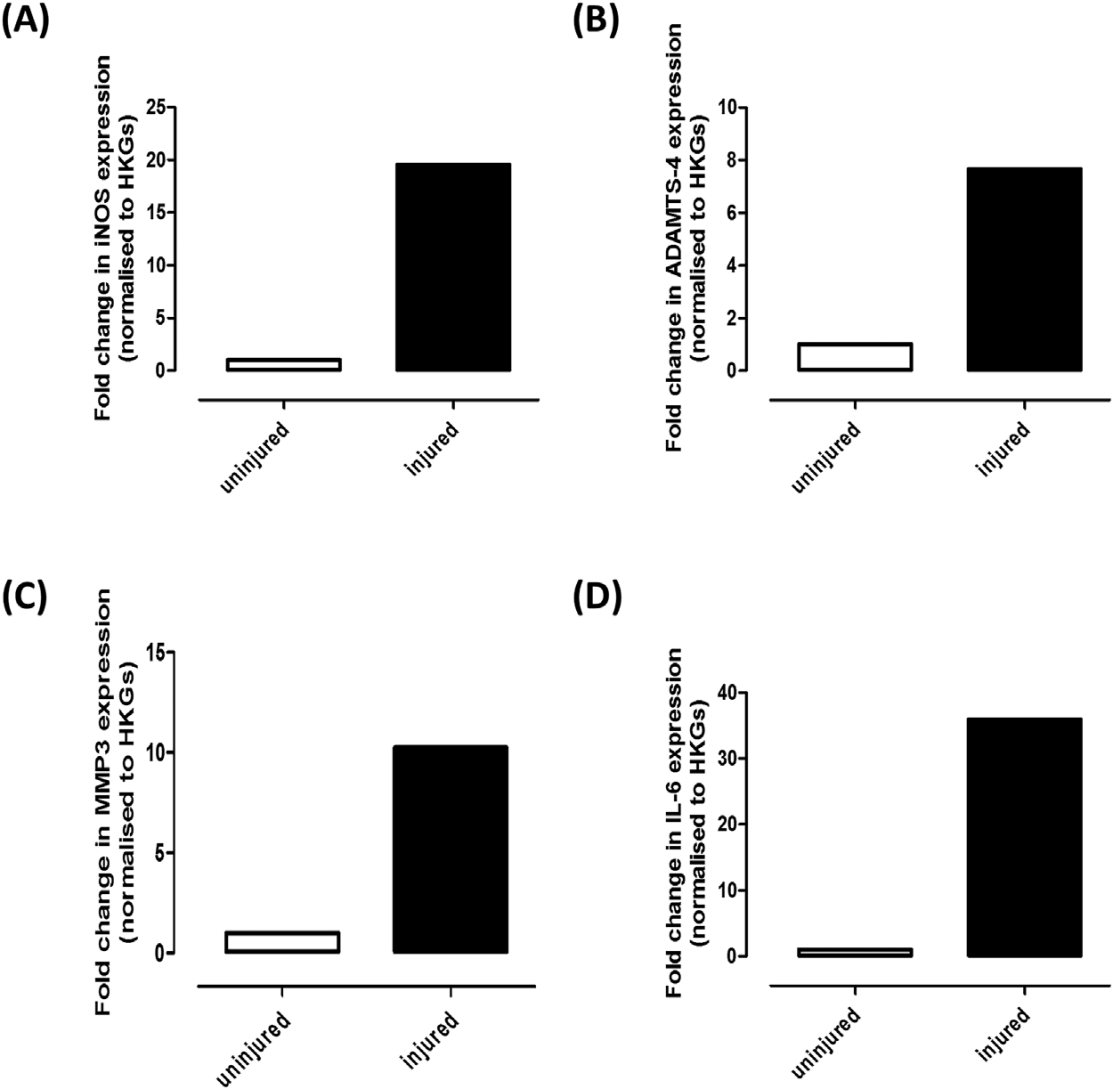
RNA pooled from the femoral condyles of nine uninjured and nine injured knees was analyzed by quantitative PCR to determine the relative expression of (A) iNOS, (B) ADAMTS‐4, (C) MMP‐3, and (D) IL‐6. Data are presented as fold change relative to uninjured knees calculated using the ΔΔC_T_ method with 18S and β‐actin as housekeeping genes (HKGs).

Femoral condylar cartilage was analyzed for transcriptional changes (Fig. 5). In injured knees, iNOS, ADAMTS‐4, MMP‐3, and IL‐6 were elevated 19.5‐fold (A), 7.7‐fold (B), 10.2‐fold (C), and 36‐fold (D), respectively at 4‐h post‐ACL rupture. There was no change in expression of ADAMTS‐5, MMP‐9, MMP‐13, TNF‐α or IFN‐γ (data not shown).

## DISCUSSION

**Figure 6:**
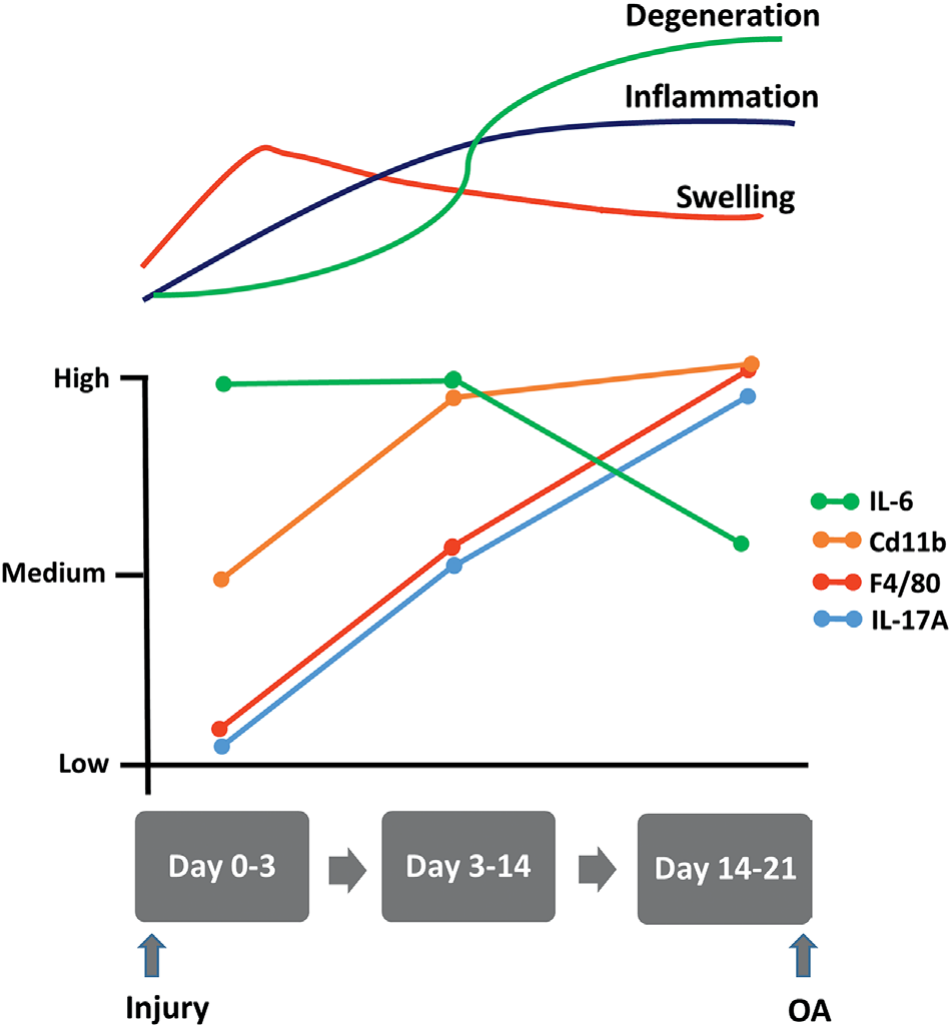
Timeline of changes that occur in inflammatory markers over the 21‐days post‐ACL rupture in our mouse model of PTOA.

We have described a non‐invasive model of PTOA whereby a single, 12N load at 1.4 mm/s caused a mid‐substance rupture of the ACL, the most commonly reported type of ACL tear.^30^ ACL rupture in humans can occur through various mechanisms but are most common in non‐contact sports following multi‐planar loading including anterior tibial shear, knee valgus, and internal tibial rotation. Our current study used a single load with a degree of rotational force to load the ACL to failure without obvious damage to the surrounding joint tissues, thus recapitulating an isolated ACL rupture injury which accounts for 18–40% of human cases.^31^, ^32^, ^33^ This is in keeping with other non‐invasive mouse models that recapitulate isolated ACL ruptures.^9^, ^24^ This non‐invasive simple to implement, highly reproducible model of PTOA displays reliable and consistent OA pathology (only six animals are required to show effects with 80% power, *p* < 0.05). The known point of injury allows disease progression to be well defined and offers a potential window of opportunity for testing early drug intervention in the absence of surgical or immune induction. It also facilitates identification of early initiating factors post‐injury and associated biomarkers, which may allow for stratification of patients to specific therapies in the future. To date, only a few studies have correlated the very early molecular, cellular and degenerative changes that occur within the mouse joint after ACL rupture^34^ [reviewed in^16^], with the majority describing the architectural changes that occur within the musculoskeletal structures.^9^, ^10^, ^17^, ^24^, ^35^ The current study provides a timeline of changes in inflammatory mediators and details the compartmental degenerative changes that result from the injury allowing the potential points of intervention to be unveiled (Fig. 6). In our model, leg swelling peaked at day‐1 and was accompanied by an acute inflammatory response over the course of the experiment. Synovial hyperplasia and inflammatory infiltrate, resulting from the rupture of the ACL, comprised a mixture of cell types that changed as the disease progressed; Cd11b^+^ cells were prominent from day‐3 whereas mature, F4/80^+^ macrophages invaded from day‐14. F4/80^+^ macrophages are closely associated with bone remodeling sites, and osteophyte formation, and when activated produce inflammatory cytokines including TNF‐α, IL‐6, and IL‐1.^36^, ^37^ By day‐14, osteophytes developed within the medial joint compartment, consistent with other groups who have shown osteophytes develop within mouse joints following ACL rupture (17 and reviewed in^18^).

IL‐6 and IL‐17 are critical in the pathogenesis of inflammatory disease^38^, ^39^ where IL‐6 induces IL‐17 production from T‐cells.^40^ After ACL rupture, IL‐6 was upregulated within the joint within 3‐days post‐injury and preceded significant increases in Cd11b^+^ macrophages, and increased inflammatory and degradative scores. This is consistent with elevated IL‐6 concentrations detected in synovial fluids in humans after joint injury.^19^, ^41^, ^42^, ^43^, ^44^ IL‐17A expression increased at days‐14 to ‐21 mirroring the abundance of F4/80^+^ cells, correlating with OA scores, particularly OA and subchondral bone components. IL‐17 up‐regulation correlates with OA progression, synergises with TNF‐α to increase articular cartilage damage, and is involved in bone remodeling.^45^, ^46^, ^47^

Gene expression analysis revealed that key inflammatory mediators (e.g., iNOS, IL‐6) and matrix degrading enzymes (ADAMTS‐4, MMP‐3) are upregulated as early as 4 h post‐injury in the femoral condyle cartilage of injured knees. A limitation of the analysis is that we only analyzed the femoral cartilage and not the rest of the joint as well as only looking at one time point. In addition, some calcified cartilage will have been included at the point of harvest. However, the data confirms that the response to injury is rapid and degeneration processes are set in motion very early on; this may reflect why interventions in patients at a later date may not be successful in preventing OA progression. In support of this, OARSI scores revealed that an increase in mean degeneration score was apparent by day‐3, with a significant increase in the OA component of the score. Disease progression was rapid and severe 21‐days after ACL rupture with changes in the medial compartment and tibial plateau occurring prior to the lateral compartment and femoral condyle. These compartmental changes mimic those of other PTOA models and may reflect a transfer of abnormal forces to the medial compartment in the absence of an intact ACL.^48^, ^49^ The degeneration occurs rapidly in our model which may not reflect the 10–12 years it takes for OA progression following injury in humans. This may be a reflection on the different biomechanics (bipedal versus quadrupedal) or may be due to differences in cartilage thickness between species. Despite this caveat, the model does allow us to study the stages of disease progression in a well‐defined timeline without the requirement of waiting decades for the disease to manifest itself in patients within the laboratory environment.

The use of a non‐invasive model of PTOA offers significant benefits over surgical models, for example, MNX. In these surgical models there are confounding inflammatory responses induced by the surgery which may obscure the pathology and prevent demarcation of early events and the testing of interventions at this time. In the current study we used the contralateral, uninjured knee as our statistical comparator which some studies suggest may be inappropriate due to altered loading on this limb following joint injury.^22^, ^50^ However, we saw no statistically significant changes in any of the outcome measures in this leg over the course of the experiment or when compared to naïve knees and thus, in line with the 3Rs we used the contralateral limb as our control. In addition to the limitations described there are a number of points that should be acknowledged regarding the use of mice as models of OA. Mice have thin articular cartilage which does not exhibit the zonal organization of human cartilage. Despite this, matrix composition is conserved between species.^51^ Mouse meniscal cartilage is often ossified which limits the benefit of examining changes that occur in this tissue; their mechanical role, however, is preserved. Murine joint biomechanics are very different to bi‐pedal humans and as such could result in divergent loading patterns and location of degenerative changes; however mechanical stresses are well conserved across species including mice.^52^ In addition, the growth plates of mice do not close which may affect the bone's response to injury and therapeutic intervention; the use of skeletally mature animals as used in the current study will go some way to overcoming this issue.

There is no clear consensus why ACL reconstruction is not an effective intervention to prevent disease progression; this may be due to irreparable damage already having occurred due to inflammatory events at the time of injury. Thus, stratification of treatments may be better aimed toward the period immediately post‐injury. The model utilized in the current study describes pathologically distinct phases of the disease increasing our understanding of the molecular and biochemical events that occur following joint injury which may translate into a targeted, therapeutic window.

## AUTHORS’ CONTRIBUTIONS

Study conception and design: SJG, VCD, DJM, and EJB. Acquisition of Data: SJG, CSB, PS, and EJB. Data analysis and interpretation: SJG, CSB, PS, VCD, DJM, and EJB. Drafting of manuscript: SJG and EJB. Revision of manuscript: All authors revised the manuscript critically for important intellectual content and approved the submitted version.

## Supporting information

Additional supporting information may be found in the online version of this article.

**Figure S1**. (A) X‐rays were taken from uninjured (UI) and injured (I) knees of (i) naïve mice, (ii) mice culled immediately after ACL rupture, and (iii) mice culled 3‐days post‐rupture. A forward translation of the tibia is apparent in injured knees following ACL rupture (•). (B) Consecutive sagittal sections were taken from the whole knees of mice that had been culled immediately following application of the 12N load, stained with Safranin O and examined for the presence of ACL damage. The sections shown are from approximately the same position within the joint from (i) uninjured and (ii) injured knees. A mid substance tear of the ACL is clearly visible (arrow).

**Figure S2**. Immunohistochemistry control sections whereby the primary antibody was replaced with (A) PBS, (B) rabbit IgG or (C) rat IgG.

**Figure S3**. Immunohistochemistry localisation of F4/80 and CD11b showing their expression in uninjured and injured knees at 3, 14 and 21‐days post‐ACL rupture. Scale bar = 20 μM. Positive cells were detected in all knees within the bone marrow but only strongly within the ACL/PCL complex of injured knees at days‐14 and −21 (yellow arrow).

**Figure S4**. Low power images of sections taken from uninjured and injured legs at day‐3, 14 and −21 stained with antibodies to IL‐6 and IL‐17A. In uninjured legs, IL‐6 was located in the cells surrounding the ACL and PCL complex (red arrows), the growth plates (white arrows) and the synovium (black arrows). In injured legs, labelling for IL‐6 was extensive throughout the ACL/PCL (red arrows) and inflammatory infiltrate (black arrows) particularly at days‐3 and −14 and within the developing osteophyte at days‐14 and −21 (*). In uninjured legs, IL‐17A was located in cartilage cells (green arrows) and growth plates (white arrows). In injured legs, IL‐17A was detected in the synovial infiltrate at all time points, and within the ACL/PLC complex, osteophytes (*) and hypertrophic cells of the meniscus at days‐14 and −21 (yellow arrows).

Supporting Table S1.

Supporting Table S2.

Supporting Table S3.
