# Supplementary figures and images for: Inflammatory and degenerative phases resulting from anterior cruciate rupture in a non‐invasive murine model of post‐traumatic osteoarthritis
Source: J Orthop Res. 2018 Mar 14;36(8):2118–27. doi: 10.1002/jor.23872 (PMC6120532; doi:10.1002/jor.23872)

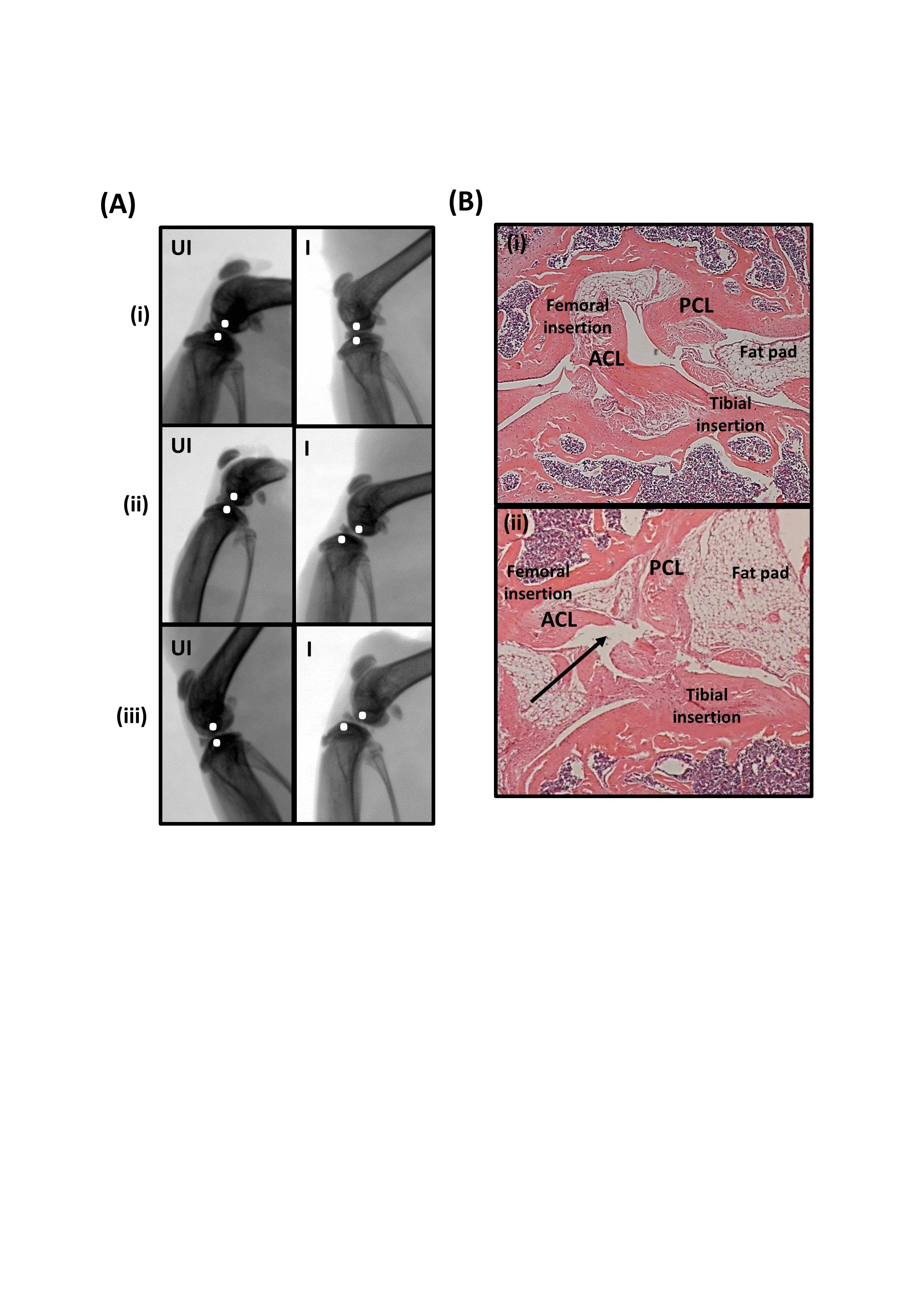

Supplement: Supplementary file 1 — Figure S1. (A) X‐rays were taken from uninjured (UI) and injured (I) knees of (i) naïve mice, (ii) mice culled immediately after ACL rupture, and (iii) mice culled 3‐days post‐rupture. A forward translation of the tibia is apparent in injured knees following ACL rupture (•). (B) Consecutive sagittal sections were taken from the whole knees of mice that had been culled immediately following application of the 12N load, stained with Safranin O and examined for the presence of ACL damage. The sections shown are from approximately the same position within the joint from (i) uninjured and (ii) injured knees. A mid substance tear of the ACL is clearly visible (arrow). [file JOR-36-2118-s001.TIF]

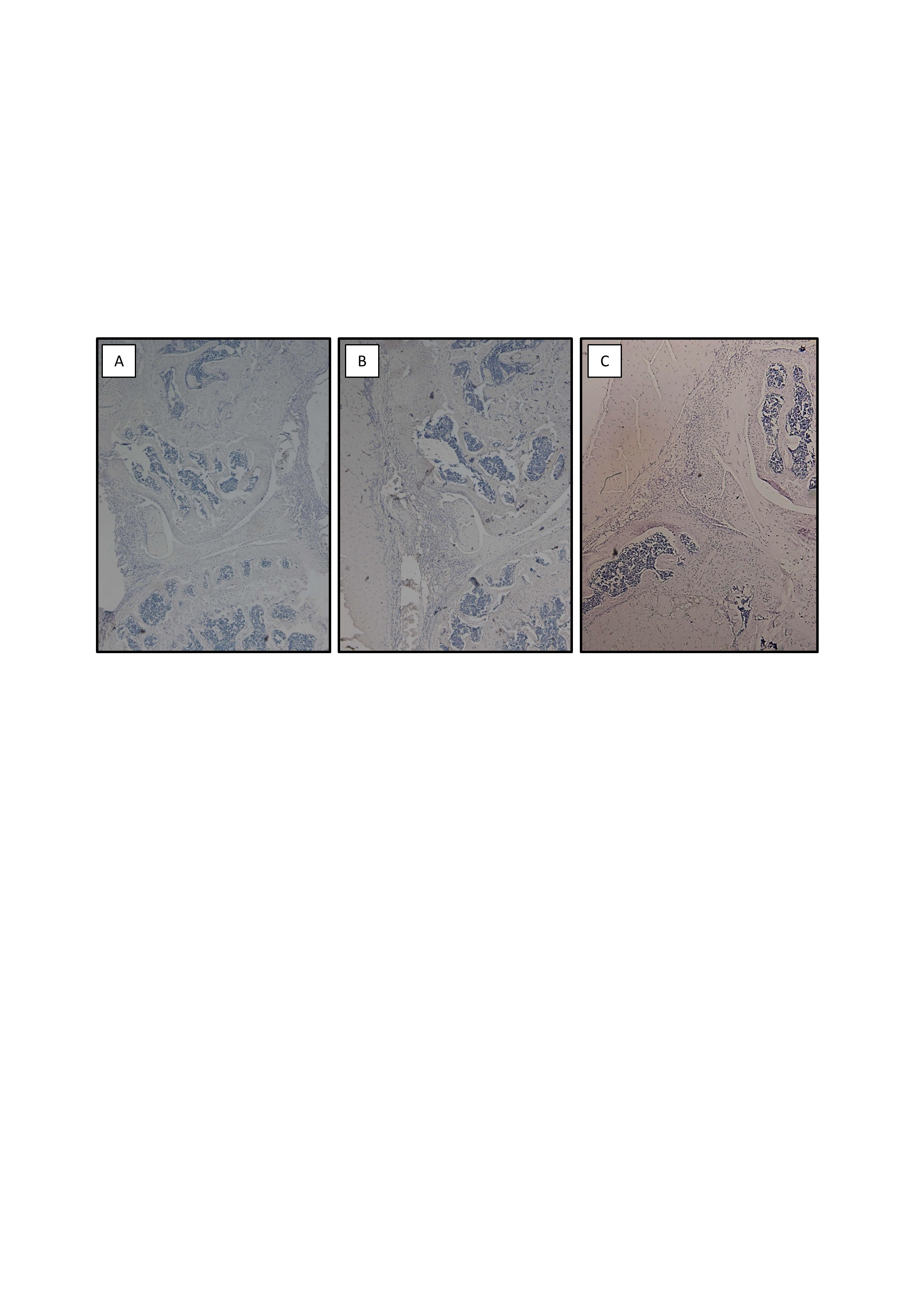

Supplement: Supplementary file 2 — Figure S2. Immunohistochemistry control sections whereby the primary antibody was replaced with (A) PBS, (B) rabbit IgG or (C) rat IgG. [file JOR-36-2118-s002.TIF]

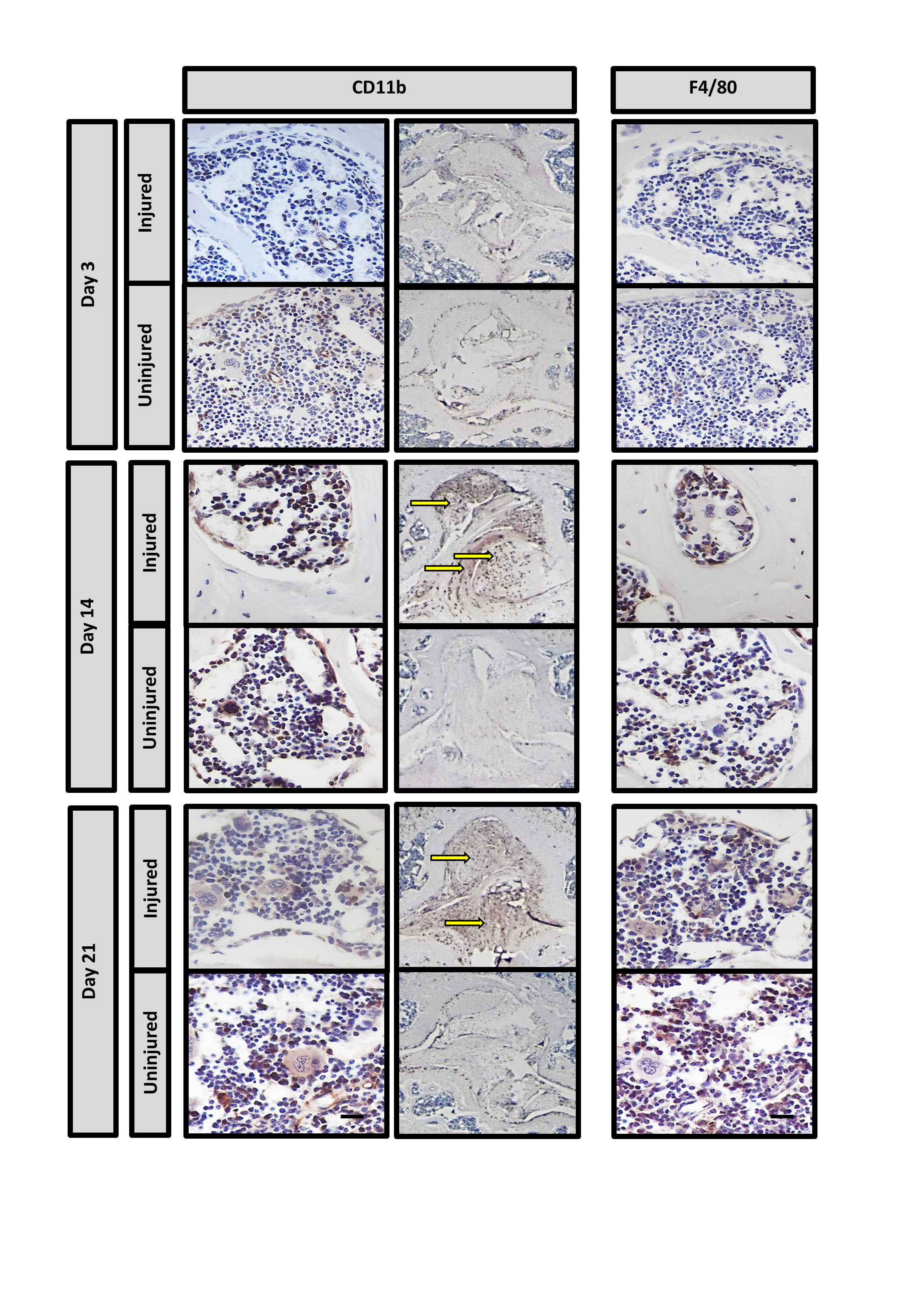

Supplement: Supplementary file 3 — Figure S3. Immunohistochemistry localisation of F4/80 and CD11b showing their expression in uninjured and injured knees at 3, 14 and 21‐days post‐ACL rupture. Scale bar = 20 μM. Positive cells were detected in all knees within the bone marrow but only strongly within the ACL/PCL complex of injured knees at days‐14 and −21 (yellow arrow). [file JOR-36-2118-s003.TIF]

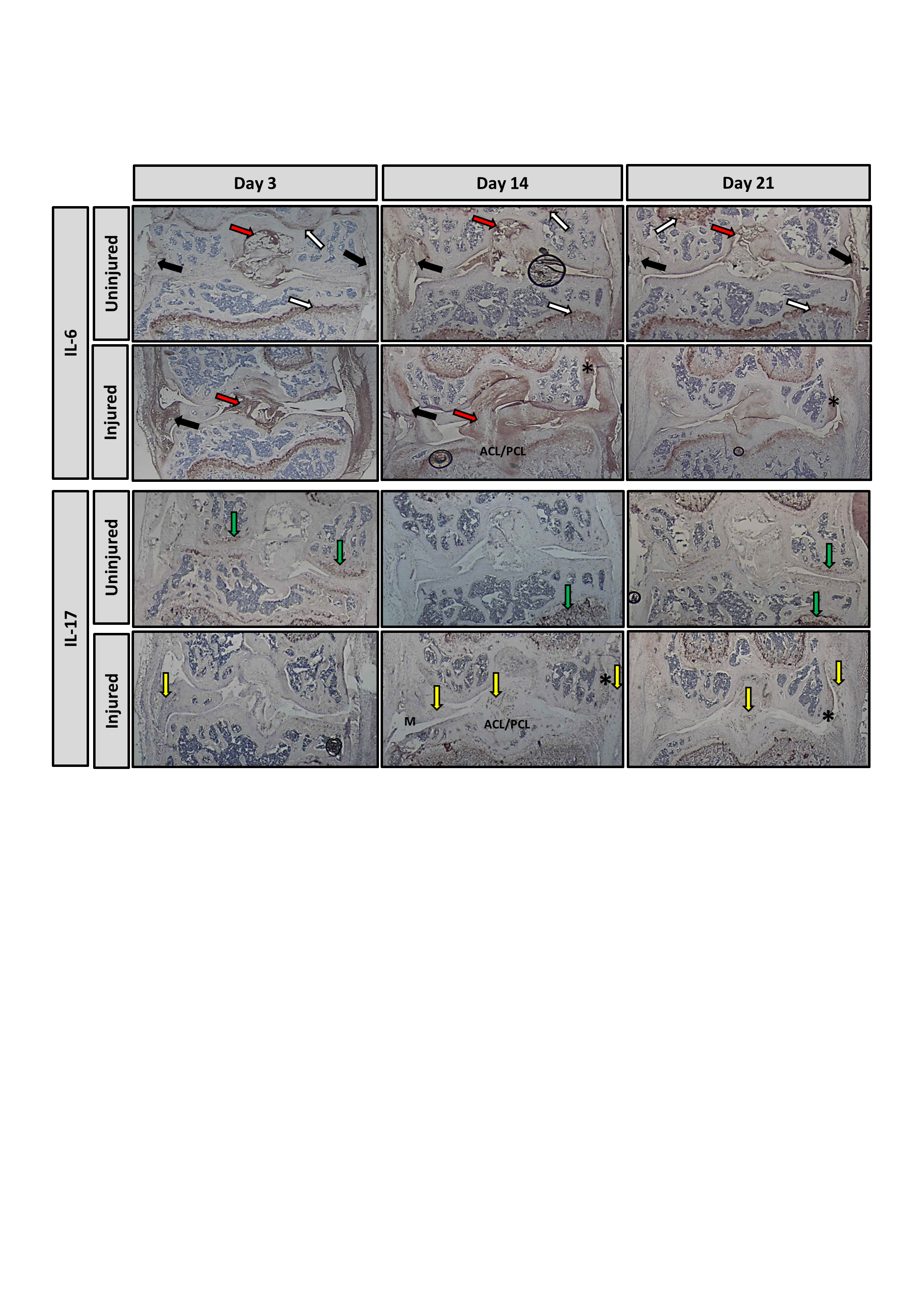

Supplement: Supplementary file 4 — Figure S4. Low power images of sections taken from uninjured and injured legs at day‐3, 14 and −21 stained with antibodies to IL‐6 and IL‐17A. In uninjured legs, IL‐6 was located in the cells surrounding the ACL and PCL complex (red arrows), the growth plates (white arrows) and the synovium (black arrows). In injured legs, labelling for IL‐6 was extensive throughout the ACL/PCL (red arrows) and inflammatory infiltrate (black arrows) particularly at days‐3 and −14 and within the developing osteophyte at days‐14 and −21 (*). In uninjured legs, IL‐17A was located in cartilage cells (green arrows) and growth plates (white arrows). In injured legs, IL‐17A was detected in the synovial infiltrate at all time points, and within the ACL/PLC complex, osteophytes (*) and hypertrophic cells of the meniscus at days‐14 and −21 (yellow arrows). [file JOR-36-2118-s004.TIF]

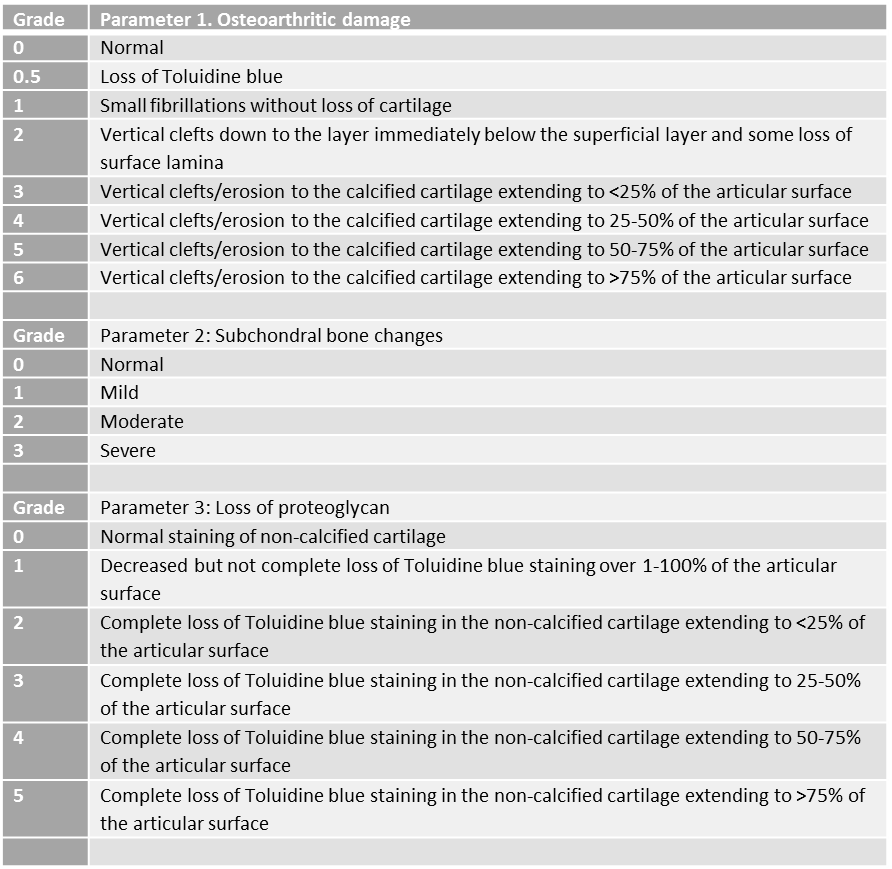

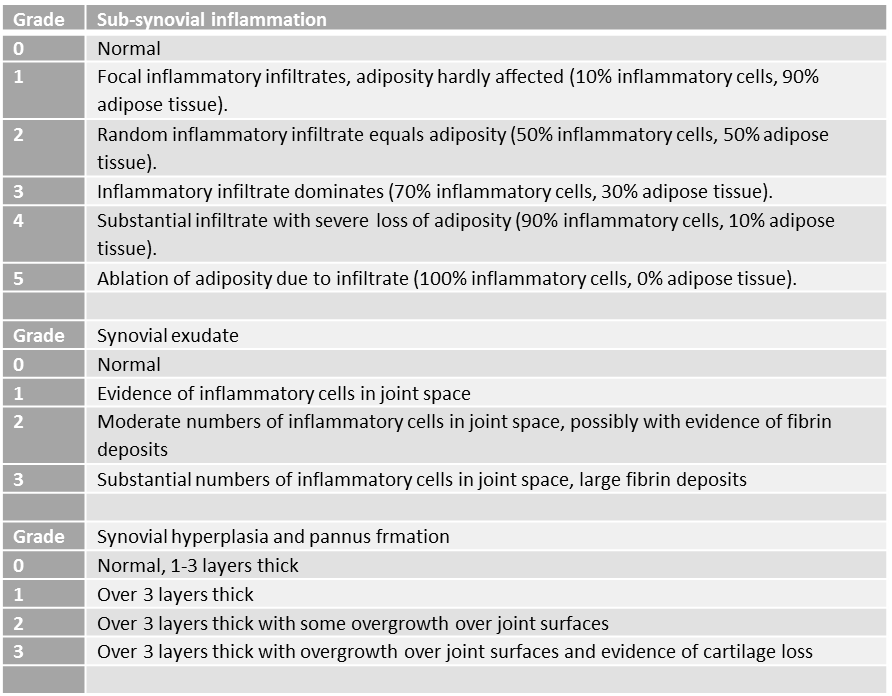


**Suppl. table 2. (A) OARSI and (B) Sub-synovial inflammation score**

**(A)**

**(B)**

Supplement: Supplementary file 6 — Supporting Table S2. [file JOR-36-2118-s006.doc]

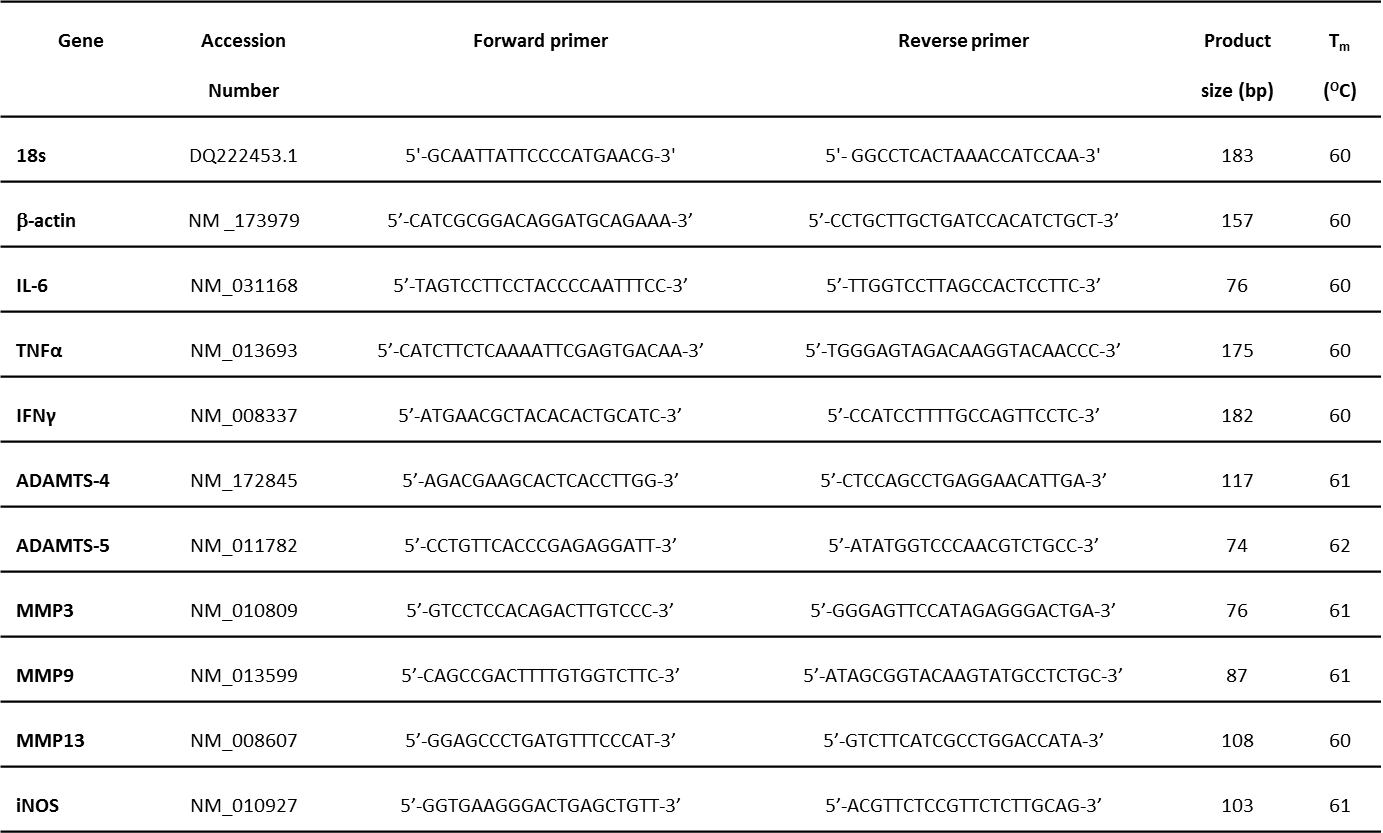


**Suppl. Table 3. Quantitative PCR primers**

Supplement: Supplementary file 7 — Supporting Table S3. [file JOR-36-2118-s007.doc]
