# Supplementary material for: Inflammatory and degenerative phases resulting from anterior cruciate rupture in a non‐invasive murine model of post‐traumatic osteoarthritis
Source: J Orthop Res. 2018 Mar 14;36(8):2118–27. doi: 10.1002/jor.23872 (PMC6120532; doi:10.1002/jor.23872)
Supplement: Supplementary file 5 — Supporting Table S1. [file JOR-36-2118-s005.doc]

| **Parameter** | **4-hours** | | **Day 3** | | **Day 14** | | **Day 21** | |
| --- | --- | --- | --- | --- | --- | --- | --- | --- |
|  | **Uninjured** | **Injured** | **Uninjured** | **Injured** | **Uninjured** | **Injured** | **Uninjured** | **Injured** |
| **Sub-synovial infiltrate** |  |  | 5 | 5 | 2 | 5 | 9 | 9 |
| **OARSI score** |  |  | 5 | 5 | 2 | 4 | 9 | 11 |
| **IL-6 immunohistochemistry** |  |  | 5 | 5 | 3 | 3 | 6 | 6 |
| **IL-17 immunohistochemistry** |  |  | 5 | 5 | 2 | 3 | 6 | 6 |
| **F4/80 and CD11b immunohistochemistry** |  |  | 1 | 1 | 1 | 1 | 2 | 2 |
| **Quantitative PCR** | 9 | 9 |  |  |  |  |  |  |
|  |  | |  |  |  |  |  |  |

**Suppl. Table 1.** Numbers of animals used for each measurement.
